# Artificial Intelligence Procedure for the Screening of Genetic Syndromes Based on Voice Characteristics

**DOI:** 10.3390/bioengineering10121375

**Published:** 2023-11-29

**Authors:** Federico Calà, Lorenzo Frassineti, Elisabetta Sforza, Roberta Onesimo, Lucia D’Alatri, Claudia Manfredi, Antonio Lanata, Giuseppe Zampino

**Affiliations:** 1 Department of Information Engineering, University of Florence, 50139 Florence, Italy; federico.cala@unifi.it (F.C.); lorenzo.frassineti@unifi.it (L.F.); antonio.lanata@unifi.it (A.L.); 2 Department of Information Engineering, Università degli Studi di Pisa, 56122 Pisa, Italy; 3 Department of Life Sciences and Public Health, Faculty of Medicine and Surgery, Catholic University of Sacred Heart, 00168 Rome, Italy; elisabetta.sforza@unicatt.it (E.S.); giuseppe.zampino@unicatt.it (G.Z.); 4 Centre for Rare Diseases and Transition, Department of Woman and Child Health and Public Health, Fondazione Policlinico Universitario A. Gemelli IRCCS, 00168 Rome, Italy; roberta.onesimo@policlinicogemelli.it; 5 Unit for Ear, Nose and Throat Medicine, Department of Neuroscience, Sensory Organs and Chest, Fondazione Policlinico Universitario A. Gemelli IRCCS, 00168 Rome, Italy; lucia.dalatri@policlinicogemelli.it; 6 European Reference Network for Rare Malformation Syndromes, Intellectual and Other Neurodevelopmental Disorders—ERN ITHACA

**Keywords:** genetic syndrome, acoustical analysis, artificial intelligence, machine learning, classification

## Abstract

Perceptual and statistical evidence has highlighted voice characteristics of individuals affected by genetic syndromes that differ from those of normophonic subjects. In this paper, we propose a procedure for systematically collecting such pathological voices and developing AI-based automated tools to support differential diagnosis. Guidelines on the most appropriate recording devices, vocal tasks, and acoustical parameters are provided to simplify, speed up, and make the whole procedure homogeneous and reproducible. The proposed procedure was applied to a group of 56 subjects affected by Costello syndrome (CS), Down syndrome (DS), Noonan syndrome (NS), and Smith–Magenis syndrome (SMS). The entire database was divided into three groups: pediatric subjects (PS; individuals < 12 years of age), female adults (FA), and male adults (MA). In line with the literature results, the Kruskal–Wallis test and post hoc analysis with Dunn–Bonferroni test revealed several significant differences in the acoustical features not only between healthy subjects and patients but also between syndromes within the PS, FA, and MA groups. Machine learning provided a k-nearest-neighbor classifier with 86% accuracy for the PS group, a support vector machine (SVM) model with 77% accuracy for the FA group, and an SVM model with 84% accuracy for the MA group. These preliminary results suggest that the proposed method based on acoustical analysis and AI could be useful for an effective, non-invasive automatic characterization of genetic syndromes. In addition, clinicians could benefit in the case of genetic syndromes that are extremely rare or present multiple variants and facial phenotypes.

## 1. Introduction

The human voice results from a complex configuration, arrangement, and coordination of the elements that make up the phonatory apparatus, the respiratory system, and the central nervous system. Therefore, abnormal neurological and anatomical features often related to genetic syndromes could alter voice production. Over the years, several works have investigated the detection of voice pathology due to benign formations (e.g., nodules and polyps), neuromuscular disorders (e.g., paralysis of the vocal cords) [1,2] and neurodegenerative diseases such as Parkinson’s disease [3,4] by using acoustical features extracted from a sustained vowel (/a/). Vocal-tract, larynx, and vocal-fold abnormalities can be identified by analyzing key acoustical parameters assessed perceptually by experienced clinicians and objectively by dedicated software. In the latter context, some of the most important parameters are [5]:

- The fundamental frequency (F0), which describes the vibration frequency of the vocal folds;
- The first formant (F1), which is related to the constriction of the anterior half of the oral cavity; the larger the cavity, the lower the F1. F1 is also raised by the constriction of the pharyngeal tract;
- The second formant (F2) (linked to tongue movements), which is lowered by posterior tongue constriction and raised by anterior tongue constriction;
- The third formant (F3), which depends on the rounding of the lips; the more this configuration is accentuated, the lower the F3;
- F0 and formants F1–F3, which are inversely proportional to the size and thickness of the vocal folds and the length of the vocal tract.

In the last two decades, acoustical analysis has been applied to patients affected by genetic syndromes such as Costello (OMIM #218040, CS), Down (OMIM #190685, DS), Noonan (OMIM #163950, NS), and Smith–Magenis syndromes (OMIM #182290, SMS), with interesting results highlighting highly irregular voices. The non-invasive semiotics of these diseases are generally determined by studying somatic traits. A promising approach to obtain a more detailed phenotype involves the use of objective acoustical analysis to identify parameters associated with individual pathological conditions.

Perceptually, low tonality and voice intensity, as well as hoarseness, are typical characteristics of adult CS individuals [6]. The rarity of this syndrome has made it particularly difficult to outline a precise acoustical profile, and no objective acoustical analysis has been carried out on these patients.

In Down syndrome, Moura et al. [7] found statistically significant differences compared to HS in the F0 for sustained vowels (/a /, /e /, /i /, and /ɔ/), as well as for formants F1–F2 and HNR measures in Portuguese-speaking children. In adults, Bunton and Leddy [8] highlighted difficulties in phonating the three cardinal vowels (/a/, /I/, and /u/), which were associated with reduced vowel space and intelligibility.

Turkyilmaz et al. [9] analyzed the sustained /a/ vowel of 11 children with Noonan syndrome using MVDP software (Kay Elemetrics Corporation, Lincoln Park, NJ, USA); no significant differences were found compared to a control group, except for the soft phonation index (SPI). Moreover, in a single case report, Wilson and Dyson [10] found vowel neutralization and nasalization in a female child.

In a study by Hidalgo et al. [11], SMS adults showed higher F0 values for the vowel /a/ than normophonic subjects, but no significant differences were found in voice disorder measures. In another paper [12], the same authors experimented with the same vocal task with SMS children, analyzing formants F1–F2 and cepstral peak prominence (CPP) [13], an important parameter for the assessment of dysphonia. Only CPP showed a significant difference between patients and controls.

These studies suggest that acoustical analysis can provide helpful information to doctors and speech therapists. However, the cited works focused on a single pathology and identified acoustical parameters that show statistically significant differences compared to the normophonic case. It is therefore important to extend this research by analyzing and comparing the vocal phenotypes of different syndromes to find significant changes that could support and speed up differential diagnosis and guide clinicians in the development of treatment or rehabilitation programs, especially for syndromes that are characterized by several variants that have yet to be discovered, such as Noonan’s syndrome [14]. Artificial intelligence represents a powerful automatic system to recognize and monitor actions and movements of daily life in elder care [15] and to analyze and distinguish images of patients diagnosed with various genetic syndromes by relying on features extracted from their facial appearance [16]. However, such a technique has never been applied to these patients’ voice and speech characteristics, even though both anatomo-physiological and statistical studies of voice quality measures have shown significant differences compared to healthy subjects. Therefore, a methodology that allows for homogeneous collection of relevant data to perform deeper statistical analysis and novel machine learning experiments could be helpful. In this work, we propose a procedure for standardizing the recording and analysis of patients’ voices affected by different genetic syndromes. Specifically, this procedure focuses on the most appropriate devices for voice recording and vocal tasks. To ensure repeatability, we also propose signal preprocessing and feature extraction steps. Furthermore, this thesis describes statistical analysis, the development of machine learning models, and performance evaluation. The validation, feasibility, and robustness of the proposed procedure were tested by applying the proposed approach to 72 patients recruited at the Fondazione Policlinico Universitario A. Gemelli (FPUG) in Rome, Italy.

## 2. Background

When performing voice analysis, it is essential to consider several aspects. In this section, we highlight the theoretical and practical basis that was considered to outline our procedure regarding recording devices, vocal tasks, preprocessing methods, feature extraction, and machine learning techniques.

### 2.1. Audio Recordings

In the guidelines provided by the Committee on Phoniatrics of the European Laryngological Society (ELS) [17], the importance of high-quality recordings is highlighted as far as both perceptual and acoustical analyses are concerned. However, no further specifications are given, which may cause difficulty in selecting an adequate recording system. Indeed, the choice of the most suitable device and its characteristics strongly depends on the application. Nevertheless, some general rules can be identified for the most appropriate microphones for different purposes [18]:

1. Flat frequency response;
2. Noise level at least 15 dB lower than the sound level of the softest phonation;
3. Dynamic-range upper limit higher than the sound level of the loudest phonation;
4. Distance between the microphone and source for which the maximally flat frequency response occurs.

For example, a cardioid microphone performs well in noisy environments for perturbation metrics. On the other hand, its short distance to the sound source may distort spectral evaluation and result in unreliable sound pressure level measurements, as pointed out by Svec and Granqvist [18]. However, such microphones can be easily miniaturized and mounted on the subject with a clip, making them particularly well-suited for children or patients with behavioral issues to avoid distraction, which could negatively affect phonation and task completion. This recording device has been successfully used to study vowel utterances of patients with Smith–Magenis syndrome [12] and Williams syndrome [19]. To reduce ambient noise, a headset with incorporated microphones has been proposed as well [20]. However, this choice strictly depends on the experimental design (i.e., Down syndrome prosody evaluation through serious games), and its use with other syndromes should be considered with caution due to its possible discomfort. Condenser microphones represent another possibility; for example, Bunton and Leddy [8] used a SHURE SM81 microphone to record the voices of Down syndome patients. Smart phones represent a promising alternative for voice recordings; several studies have reported their efficiency, portability, and cost-effectiveness in collecting pathological voices [21,22,23], although they must be used with caution in noisy environments and in terms of distance and inclination with respect to the radiation source. Manfredi et al. [24] tested two smart phones at the extremes of the commercial price range, which were found to perform similarly in voice acquisition, suggesting that almost any smartphone-integrated microphone could be used to reliably record audio signals for acoustical analysis purposes. Cavalcanti et al. [25] performed a similar analysis, finding that smartphones seem not to alter most acoustical properties as compared to professional microphones, except for the harmonic-to-noise ratio (HNR) and cepstral peak prominence (CPP). These results partially agree with those reported in a study by Glover and Duhamel [26], where noise measurements were significantly different when comparing audio samples from smart phones and digital voice recorders. However, such differences may have been caused by incorrect positioning of recording devices and the small number of participants. As Cavalcanti et al. [25] state, the highly dynamic smartphone industry, the lack of standardization, and the fact that companies usually do not disclose microphone characteristics limit the effective usability of smartphones. Nevertheless, their ubiquity and ease of use offer a relevant opportunity to monitor the voice quality for longitudinal evaluation and during daily activities. Sound-treated booths are advisable, especially when using smart phones. However, they can make the acquisition process slower and more complex, undermining the advantages of using a smart phone.

### 2.2. Vocal Tasks

The evaluation of voice condition is typically based on two types of utterances: sustained vowels and running speech. In this work, we refer to [5] for vowel symbols. Several papers have highlighted that certain pathologies can be more easily described and identified when examining specific tasks. For instance, Hidalgo-De la Guia et al. [19] required the utterance of the /a/ vowel to favor a less forced phonation in patients with neuromotor deficits due to the stable tongue and jaw position. Frassineti et al. [27] proved that adding /I/ and /u/ phonations improved pathology detection. The /e/ and /o/ vowels can be used as well. for example, Suppa et al. [28] performed acoustical analysis of a sustained /e/ to detect Parkinson’s disease in elderly patients; however, these utterances are more sensitive to dialects, and therefore, results may be less reliable. Instead, /a/, /I/, and /u/, usually referred to as cardinal or corner vowels, are characterized by a well-defined vocal tract configuration and remain stable during articulation, which makes them substantially independent of dialectal and even linguistic diversity [29].

Running speech could provide further information to describe voice quality, as some aspects are highlighted in a voiced context or after a glottal closure. According to the European Laryngology Society (ELS), it is essential that running speech in the form of a single sentence or a short, standardized passage be characterized by constant voicing and not contain fricatives. This reduces possible biases in the computation of noise-level metrics due to articulation noise and better highlights the habitual fundamental frequency during speaking. As suggested by Gomez-Garcia et al. [30], a coarticulation task should also be included to evaluate the influence of the preceding and succeeding acoustical units on the current unit under analysis. For instance, Alpan et al. [31] successfully employed this utterance to predict perceptual scores of the GRBAS scale with acoustical parameters.

The Società Italiana di Fonologia e Laringologia (SIFEL) considers the singing voice to evaluate a different aspect of speech production [17]; it does not entirely reflect daily conversational performance, but since voice is used at a higher functional level, it can provide an interesting insight into vocal properties, even in non-professional singers or actors. Seok et al. [32] demonstrated that adding such a task to their voice evaluation protocol allowed for better assessment of vocal properties before and after thyroid surgery, helping to monitor postoperative voice changes and improving the assessment of subjective voice discomfort.

### 2.3. Preprocessing of Audio Samples

Speech and voice signals may undergo several preprocessing techniques to enhance feature extraction. One of the most used techniques is inverse filtering. In voice pathology detection, the vibratory dynamics of the vocal folds can be analyzed, removing the influence of vocal-tract resonances [33,34]. However, when dealing with genetic syndromes, vocal-property alterations may not be uniquely associated with biomechanical factors of the vocal folds but also with several morphological anomalies that affect the vocal tract. Examples include laryngomalacia, redundant nasal tissue, and hypopharyngeal veil collapse for CS [6,35]; arytenoid cartilage enlargement and pharyngeal constriction for DS [20,36]; ogival palate and tongue malformations for NS [37]; and velopharyngeal insufficiency for SMS [38]. Therefore, filtering to reduce background or convolutional noise is discouraged in our procedure, as it could remove important information about irregularities or turbulence connected with the pathologies themselves [30]. Thus, more appropriate tools could consist of voice/unvoiced and/or silence detectors, which might allow for more efficient identification of the segments of the recording in which vocal-fold vibration occurs [39,40]. This approach can be applied to sustained vowels as well.

### 2.4. Acoustical Feature Extraction

The aim of characterizing voice signals is to extract features to describe the properties of the pathological groups under examination. Gomez-Garcia et al. [30] pointed out that identifying voice impairments is a difficult task, since certain phenomena typically associated with vocal disorders (i.e., aperiodicity) can be inherent to physiological phonation processes. Thus, it is important to implement techniques to obtain a large number of features to maximize the probability of finding a range of metrics capable of separating normophonic and pathological subjects and/or differentiating pathologies. In recent years, several methodologies have been proposed and implemented to improve feature extraction; however, the interpretability of the results must be considered, especially when exploratory analyses are performed. The open-source BioVoice tool performs objective acoustical analysis [41] in both the time and frequency domains. The number, length, and percentage of voiced and unvoiced segments (V/UV) are detected in the time domain. In the frequency domain, the fundamental frequency (which measures the vibration of the vocal folds in Hz (F0)), formant frequencies (in Hz, which are related to the configuration of the articulators along the vocal tract (F1–F3)), noise level (normalized noise energy (NNE), in dB), and jitter (in %) are estimated. NNE ranges from 0 dB downwards. Thus, the higher the noise level, the closer its value is to 0 dB. For F0 and each formant, the mean, median, standard deviation, maximum, and minimum values are calculated. Moreover, the power spectral density (PSD) is computed in the frequency range of each category (newborn, child, adult female, adult male, and singer), and normalized with respect to its maximum value; this allows for comparison among different PSDs. Excel tables and pictures are automatically saved in devoted folders (one for each recording). The BioVoice dropdown menu allows the user to choose the gender, age, and type of emission without requiring any manual setting to be selected. This greatly simplifies its usage by non-expert users, automatically adjusting the frequency ranges for F0 and formant estimation with respect to other commonly used software, such as PRAAT [42].

### 2.5. Machine Learning

The automatic assessment of voice quality based on machine learning (ML) represents a well-established strategy that typically relies on supervised learning techniques such as k-nearest neighbors (KNN), support vector machines (SVM), and random forest (RF) [1,30]. Statistical analyses of the voice properties of genetic syndromes have already highlighted significant differences in phonation and articulation. When focusing on prediction rather than inference, it is crucial to develop a tool capable of generalizing the underlying pattern of training data to identify new observations. ML is suitable for this task, and several studies have demonstrated that such methods can separate data even if they do not show statistically significant alterations [43,44]. Moreover, ML requires few assumptions about the data-generating systems, as opposed to statistical analysis, where possible violations of the assumptions can lead to unreliable results. In a previous study, we demonstrated that machine learning algorithms can effectively discriminate between the same four genetic syndromes with an overall accuracy < 50% [27]. However, feature extraction was performed with PRAAT, and healthy subjects were not considered. These promising results need further examination.

## 3. Materials and Methods

**Figure 1:**
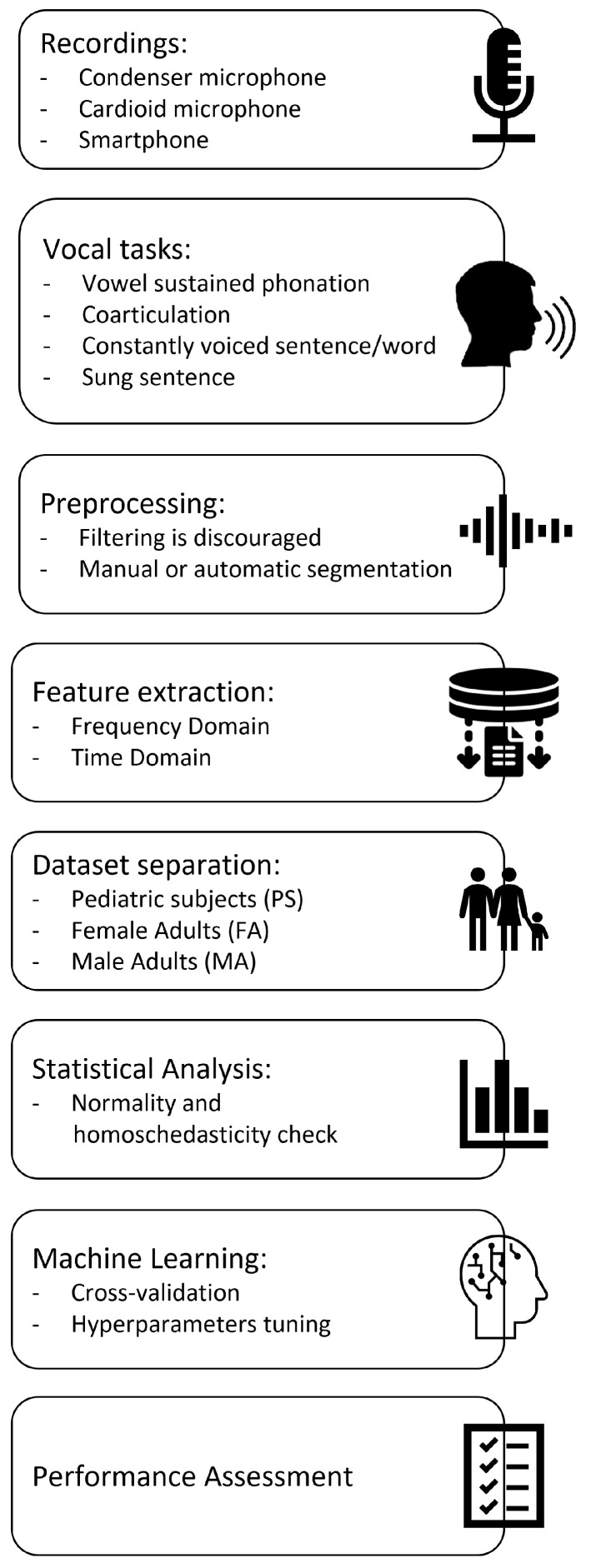
Procedure pipeline.

Figure 1 displays and summarizes the proposed procedure. Details are given in the following subsections: Recordings, Vocal tasks, Preprocessing of audio samples, Acoustical analysis, Machine Learning, and Statistical Analysis. Procedure assessment is discussed in the subsection named Procedure validation.

### 3.1. Recordings

Taking into account the pathological subjects under study, our procedure suggests the use of a smart phone, as it allows for the collection of a large number of recordings quite easily and quickly. Only one smartphone model should be used for all acquisitions to ensure experimental repeatability and uniformity of the recordings. This device should be kept 15 cm from the mouth at a 45${}^{\circ }$ inclination to reduce lateral distortions [45]. Background noise should be <50 dB, and subjects must speak with conversational tone and intensity.

### 3.2. Vocal Tasks

Factors like age, scarce cooperation, language deficits, and cognitive disorders pose a challenge to the feasibility of running speech and singing tasks in patients affected by genetic syndromes, since they are typically characterized by severe cognitive and behavioral impairments. Despite such difficulties, our procedure consists of three repetitions of the following items (Italian language):

- List of numbers from 1 to 10;
- Word /aiuole/ (IPA transcription: «a’jwɔle»; English translation: «flowerbeds»);
- Vowels /a/, /e/, /I/, /o/, and /u/, sustained for at least 3 s;
- Sentence “io amo le aiuole della mamma” (IPA transcription: «’io ‘amo ‘le a’jwɔle ‘del:a ‘mam:a»; English translation: “I love mother’s flowerbeds”);
- Sung sentence “Fra Martino campanaro, dormi tu” (Italian version of the first sentence of the well-known European traditional song Frère Jacques).

Three repetitions are required to account for biological variability and obtain (usually by averaging) more reliable parameters [46]. However, this is not always possible, depending on the severity of the pathology.

In this work, acoustical analysis is performed on /a/, /I/, and /u/ utterances only.

### 3.3. Preprocessing of Audio Samples

In our procedure, we suggest selecting only the central part of the signals to obtain more reliable acoustical measures, as they correspond to the “steady-state” part of the recording. Such a selection has also been reported in the literature [25]. Manual segmentation was carried out for validation of the procedure using Audacity software [47].

### 3.4. Acoustical Analysis

Thirty-seven parameters were extracted from audio samples with BioVoice, as listed in Table 1, along with their meanings. Some metrics were ignored because the syndromes we considered are usually not characterized by spasmodic muscle contraction or frequent voice breaking.

**Figure 2:**
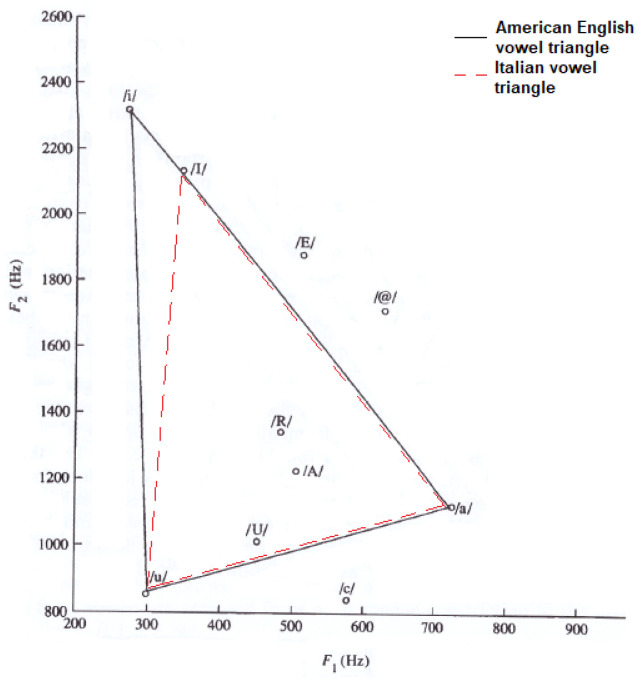
American English and Italian vowel triangles. /I/ is a cardinal vowel in Italian. /A/, /U/, /R/, /E/, /c/ and /@/ represent non-cardinal vowels in both languages.

To carry out a more detailed voice analysis, we also included articulatory parameters, which are related to the so-called vowel triangle. Figure 2 displays the American English vowel triangle [5] and the Italian vowel triangle, which refers to adult males. The only slight difference concerns the vowel /I/ (Italian), with a mean F2 value about 300 Hz lower than for the American English /i/. This difference is taken into account in this paper).

The vowel space area (VSA, Equation (1)) measures the vowel triangle area, quantifying the articulatory ability [48].

(1) $$VSA=\frac{|(F1I\times (F2a-F2u)+F1a\times (F2u-F2I)+F1u\times (F2I-F2a)|}{2}$$

The formant centralization ratio (FCR) represents a normalization procedure conducted to obtain an acoustical parameter that maximizes dysarthria detection and minimizes intervariability [49]. It is expressed as:

(2) $$FCR=\frac{F2u+F2a+F1I+F1u}{F2I+F1a}$$

Formant ratios, as proposed by Shapir et al., are other important parameters to evaluate tongue movements and articulatory capabilities [50].

(3) $$F-ratio_{aI}=\frac{F1a}{F1I}$$

(4) $$F-ratio_{au}=\frac{F1a}{F1u}$$

(5) $$F-ratio_{Iu}=\frac{F2I}{F2u}$$

In particular, Equations (3) and (4) are sensitive to vertical tongue movements, and Equation (5) is sensitive to horizontal movements.

### 3.5. Dataset Separation

Previous studies have highlighted that analyzing male and female voices together leads to less reliable results [27], mainly because of the different sizes and shapes of the phonatory apparatus. Therefore, in this work, the database was split into three groups: pediatric subjects (i.e., individuals < 12 years of age), female adults, and male adults, as denoted by the acronyms PS, FA, and MA, respectively.

### 3.6. Machine Learning

Our procedure suggests using ML techniques to develop classifiers based on objective acoustical features to distinguish four pathological classes (Costello, Down, Noonan, and Smith-Magenis syndromes) and normophonic subjects. Three models were developed for the PS, FA, and MA groups. Specifically, in this work, KNN, SVM, and RF classifiers are implemented. K-fold cross validation was performed with k = 10. Bayesian optimization was carried out to find the best model hyperparameters that maximize global accuracy, with 30 iterations for KNN and 60 iterations for SVM and RF [1]. In our study:

1. For the KNN classifier, between 2 and 27 neighbors (k) were evaluated. The considered distance metrics were “cityblock”, “Chebyshev”, “correlation”, “cosine”, “Euclidean”, “Hamming”, “Jaccard”, “Mahalanobis”, “Minkowski”, “seuclidean”, and “Spearman”. The distance weight was selected among “equal”, “inverse”, and “squared inverse”.
2. For the SVM classifier, coding was selected between “one vs. one” and “one vs. all”. The box constraint and kernel scale were evaluated between 10${}^{-3}$ and 10${}^{3}$. The kernel function was set as Gaussian.
3. For the random forest, the minimum number of leaves was selected among 2 and 27; the maximum number of splits was selected among 2 and 27; the split criterion was selected among “deviance”, “gdi”, and “twoing”; and the number of variables to sample was selected between 1 and 55.

All ML experiments were conducted in MATLAB^®^ 2020b (The MathWorks, Inc., Natick, MS, USA). A code was developed for each class to compute recall, specificity, precision, F1 score, accuracy, and area under the curve (AUC). Global accuracy was determined as well. In conclusion, for each cardinal vowel, the first 24 parameters listed in Table 1, as denoted by (+), plus 5 articulatory parameters (Equations (1)–(5)) were considered, for a total of 77 features. We remark that the details provided here in points 1–3 allow for experimental repeatability, but they do not represent the unique procedure for future works. Indeed, with a larger dataset or when considering additional syndromes, other models (including deep learning techniques) and hyperparameter tuning strategies should be tested.

### 3.7. Statistical Analysis

In addition to ML methods, a statistical analysis was performed to understand whether the BioVoice acoustical parameters allow for the detection of significant differences among syndromes and if these results align with the literature. To find the most appropriate statistical test, Shapiro–Wilk and Lèvene tests were applied to check normality and homoscedasticity, respectively. The SPSS tool (IBM Corp. Released 2021. IBM SPSS Statistics for Windows, Version 28.0. Armonk, NY, USA: IBM Corp) was used. Based on the outcome, a parametric one-way ANOVA or a non-parametric Kruskal–Wallis test was considered to carry out a multivariate analysis of the acoustical features. The α-level of significance was set equal to 0.05. Post hoc analysis considered t-tests with Tukey correction or Dunn–Bonferroni tests.

### 3.8. Procedural Validation

Fifty-six patients were recruited at Fondazione Policlinico Universitario Gemelli (FPUG), Rome, Italy. Genetic syndromes involved in this study are: Down syndrome (13 subjects), Costello syndrome (10 subjects), Noonan syndrome (17 subjects), and Smith–Magenis syndrome (16 subjects). Data from sixteen healthy subjects were also collected to make up the control group. Inclusion criteria for the control group were: absence of voice pathologies and acute or chronic inflammation of the airways (such as rhinosinusitis or asthma).

A Huawei 10 Mate smart phone was used for the recordings. Acquisitions were performed in empty rooms where only the experimenter and the patient were present (in the case of minors, a parent or a tutor was also present, and they were required to stay silent). All items of the SIFEL protocol described in [51] were recorded, but in this exploratory analysis, only those concerning the three sustained vowels (/a/, /I/, and /u/) are considered to obtain statistical results comparable with those reported in the literature. Moreover, these utterances were chosen because they were the most numerous, as they represent a relatively easy task, even for patients with severe cognitive or behavioral impairments. Recordings were acquired during regularly scheduled medical visits at the FPUG. This means that some subjects were monitored longitudinally; therefore, multiple audio files were collected. Although the measures are not strictly independent, we selected recordings with at least a 1-year interval between acquisitions to find a compromise between reaching an adequate number of data to perform robust statistical analysis and classification experiments and obtaining independent-like measures (especially in the case of pediatric subjects). This approach was necessary, as some of the considered diseases are characterized by extremely low prevalence, e.g., 1:500,000 live births for CS [35]. Data were treated anonymously, and informed consent was obtained from each participant or their parents/tutor in the case of minors. Table 2 shows the mean, standard deviation (in parentheses), and number of recordings (in square brackets) of the considered genetic syndromes and control subjects in each group.

A custom code was developed to guarantee classifier generalization capabilities for ML experiments. Specifically, the code assigns the same indexes to all available recordings of a single participant so that during the data split, they are strictly included in either the training or validation set. This approach allows classifiers to recognize participants’ identity rather than their pathology, reducing possible data leakage.

## 4. Results

None of the groups achieved a positive result on the normality test; therefore, only the Kruskal–Wallis test and post hoc analysis with a Dunn–Bonferroni test were performed. Table 3, Table 4 and Table 5 report the H statistic and *p* value only for the acoustical parameters that showed significant differences for the PS, FA, and MA groups, respectively. An acoustical parameter able to discriminate between the normophonic class and one (or more) genetic syndrome is marked with *, whereas for a separation across two (or more) pathological classes, (${}^{\#}$) is used. The details of multiple comparisons are reported in Appendix A, Appendix B and Appendix C for the PS, FA, and MA groups, respectively.

Table 6 shows the performance of machine learning classifiers for the PS, FA, and MA groups. It displays the mean value of the evaluation metric across the 10 cross-validation folders and the standard deviation (std). For pediatric subjects, a KNN model with k = 2, distance metric = city block, and weight = equal was obtained. An SVM model with box constraint = 515 and kernel scale = 11 was identified for female adults. The best model for the male adults was an SVM model with box constraint = 526 and kernel scale = 13.

**Figure 3:**
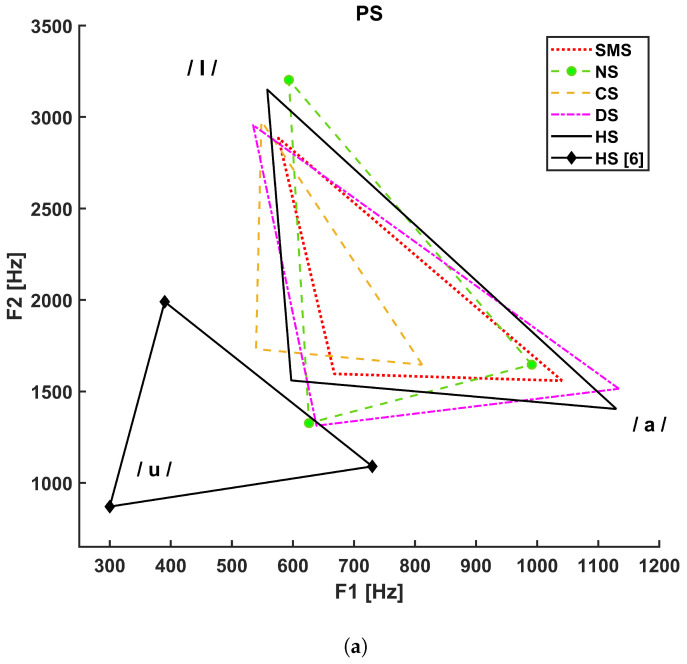

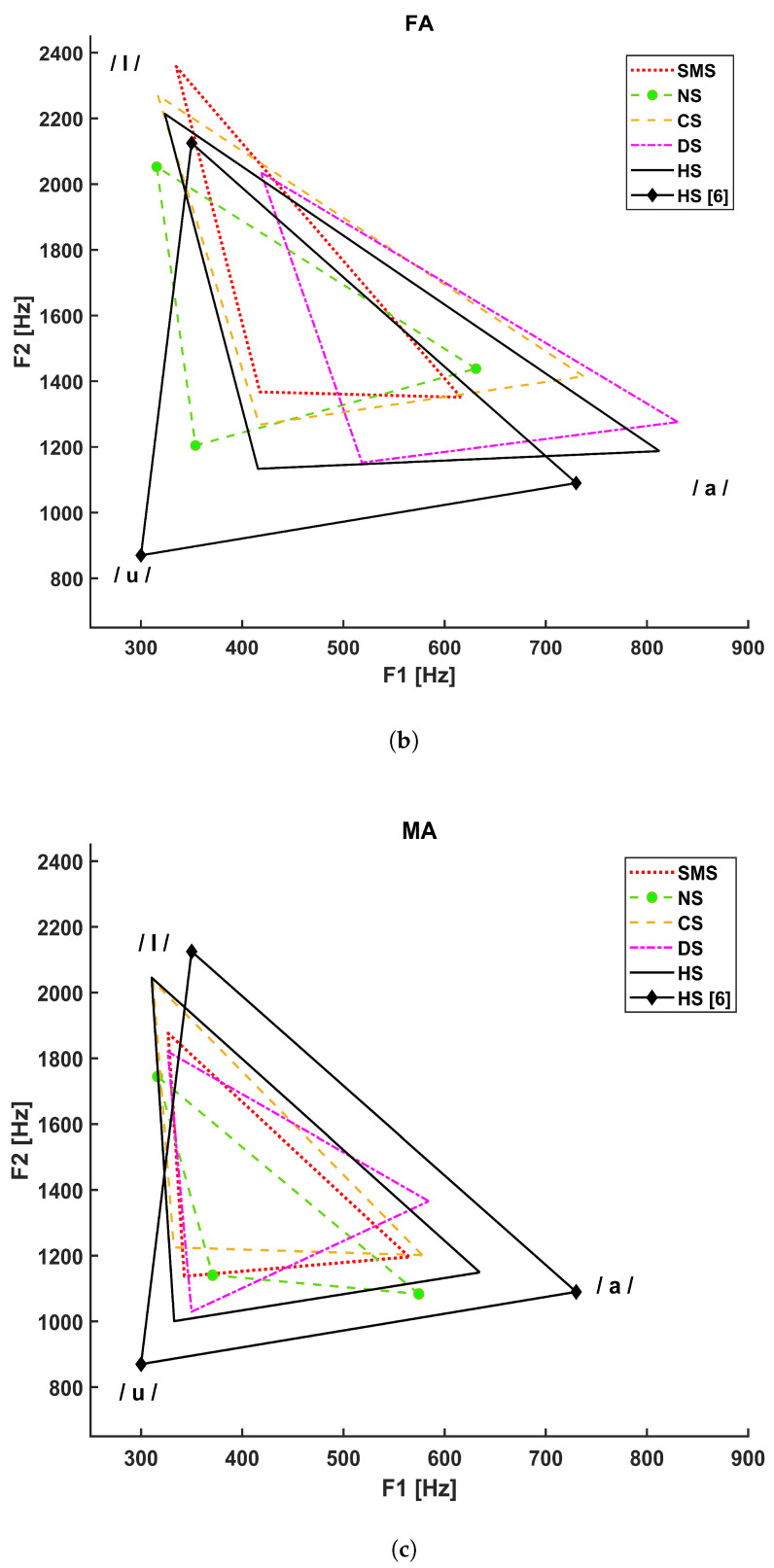
Vocalic triangles by group. (**a**) Vocalic triangle of pediatric subjects. (**b**) Vocalic triangle of adult female subjects. (**c**) Vocalic triangle of adult male subjects.

Figure 3 shows the vocalic triangles for the PS (a), FA (b), and MA (c) groups. The solid line refers to the healthy subjects considered in our study, and the solid line with diamond markers represents the vocalic triangle reported in [5], whereas:

- The dotted line refers to SMS patients;
- The dashed line with circle markers refers to NS patients;
- The simple dashed line refers to CS patients;
- The dash–dotted line refers to DS patients.

The Italian reference triangle shown in Figure 2, as represented by a solid line with diamond markers in panels A, B, and C of Figure 3, is added to compare the general results (healthy adult male subjects) to those related to our cohort of healthy subjects that differ in terms of age and gender.

**Figure 4:**
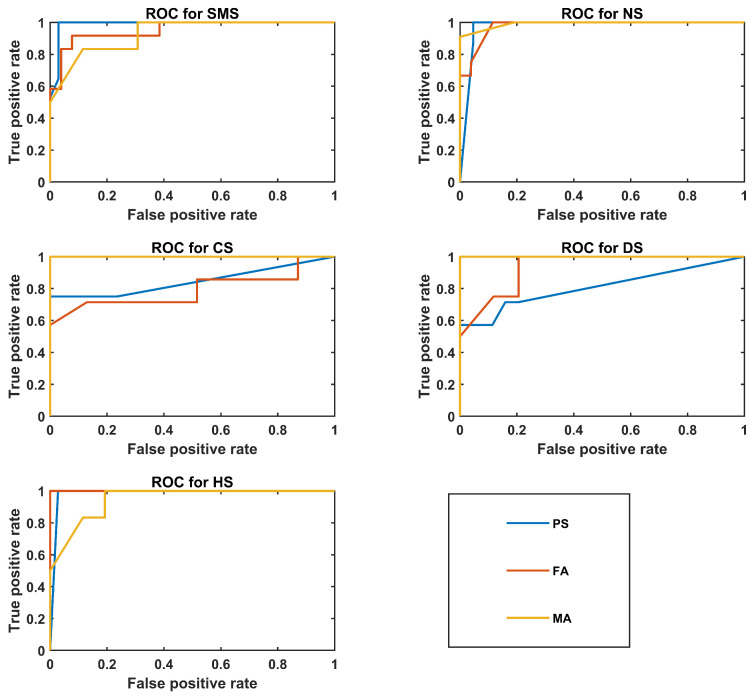
ROC plots for the best classifier of the PS, FA, and MA groups.

Figure 4 shows ROC plots for the best PS, FA, and MA classifiers.

## 5. Discussion

This paper proposes a detailed procedure for assessing the voice characteristics of patients affected by genetic diseases. It was developed according to the general guidelines provided by otolaryngological societies and associations and by reviewing literature articles on voice analysis and automatic voice quality assessment. This is the first attempt to standardize the acquisition, analysis, and classification processes of voice samples of subjects affected by genetic syndromes. Acoustical analysis represents a promising, non-invasive approach in this clinical field, and this work aims to establish ground rules for uniform and comparable results. A Huawei Mate 10 Lite (RNE-L21) smart phone was used for the recordings. Although rigorous, the proposed procedure is easily adaptable to other pathologies. Moreover, this procedure might also be applied to languages other than Italian, considering specific vocal tasks. Being an exploratory analysis, we validated it with statistical analysis and machine learning techniques and reported the outcome. Age range and gender were taken into account, which allowed us to obtain more reliable acoustical parameters. Voice properties were compared between healthy and pathological subjects and among genetic syndromes. Specifically, we considered Costello (CS), Down (DS), Noonan (NS), and Smith-Magenis (SMS) syndromes. The results are discussed in this order.

Concerning F0-related parameters, CS pediatric subjects did not show any statistically significant difference in acoustical parameters with respect to either healthy subjects or patients except, for F0 std /a/, which could reflect a lower ability to sustain vowel emission with respect to SMS patients due to generalized hypotonia or neck-muscle spasticity [52]. Articulation deficits were highlighted by the vowel triangle (shrunk and left-shifted diagram in Figure 3a), which may depend on detectable deformations of the vocal tract such as an ogival palate, macroglossia, hypopharyngeal velum laxity, and supraglottic stenosis [52]. These signs, as well as pharynx structural malformations, might cause difficulties in tongue movements. Statistical analysis detected significant differences in formant ratios (related to tongue motor ranges) and articulatory measures, e.g., the F ratio${}_{aI}$ with respect to DS (*p*-value = 0.006) and HS (*p*-value = 0.014) and FCR with respect to NS (*p*-value = 0.021), DS (*p*-value = 0.015), and HS (*p*-value < 0.001).

Vocal instability and noise metrics computed for /I/ showed significant differences in the FA CS group: jitter with respect to DS (*p*-value = 0.018) and NNE with respect to NS (*p*-value = 0.026). The latter finding agrees with the perceptual evaluation of the CS voice, which is defined as hoarse [6]. Hypotonia constraints of lips and tongue movements, especially in reaching their limit positions, and pharyngeal space reduction due to macroglossia could be the reason for significant differences in F2 mean /a/ and F2 max /a/ with respect to the control group (*p* value = 0.031 and *p* value = 0.005, respectively).

In adult CS males, statistical analysis showed differences concerning articulation, specifically with respect to NS (*p*-value = 0.044) for F2 min /a/ and with respect to HS (*p*-value = 0.024) for F2 mean /u/, which is also supported by the vowel triangle shown in Figure 3c. This could be related to structural alterations of the posterior fossa, which can cause dysarthria [53], macroglossia, or generalized hypotonia. This medical evidence also relates to a significant difference in F3 min /u/ with respect to HS (*p*-value = 0.023).

In the DS PS group, unlike the results reported by Moura et al. [7], the F0 of vowels and jitter did not significantly differ from the HS group. Such a discrepancy could be related to different spoken languages (Brazilian Portuguese in [7]), the size of the sample (The authors of [7] applied acoustical analysis to a group of patients ten times larger than the one of this study), and the software used for acoustical analysis (PRAAT [42]). In a review by Kent [36], it was also stated that voice impairments with neurologic origin cause large variability in results, especially when evaluating F0 and its perturbations. As far as formant analysis is concerned, multiple comparisons showed statistical differences with respect to CS in F1 mean /a/ (*p* = 0.002) and VSA (*p* = 0.015) and with respect to NS in F2 max /I/ (*p* = 0.021). These could be related to larger tongue dimensions, which affect tongue movements and modify vocal tract resonances.

Multiple comparisons in for DS adult females showed significant differences for jitter /I/ with respect to CS (*p* value = 0.018) and NS (*p* value < 0.001) and for jitter /u/ with respect to CS (*p* value = 0.037) and HS (*p* value = 0.038). Articulation problems, which are still present in adults, determine significant differences in F1 mean /u/ and F1 min /a/ with respect to NS (*p* value = 0.001 and *p* value = 0.028) and F2 max /u/ with respect to SMS (*p* value = 0.003).

In the MA DS group, post hoc analysis detected significant statistical differences for FCR with respect to HS (*p*-value = 0.007), for F2 mean /a/ with respect to NS (*p* value < 0.001) and HS (*p*-value = 0.015), for F2 mean /I/ with respect to HS (*p*-value = 0.004), and for F3 mean /a/ with respect to NS (*p*-value = 0.008) and HS (*p*-value = 0.004). Neurologic abnormalities located in the low temporal regions of the motor cortex could be the reason for these results.

For NS pediatric subjects, generalized low muscular tone, which tends to make lateralization and protrusion of the lips and tongue difficult and limits jaw opening, might explain statistical differences in F1 min /a/ with respect to HS (*p* = 0.024), in F2 mean /a/ with respect to HS (*p* = 0.001), in F2 mean /I/ with respect to SMS (*p* = 0.001), and F ratio${}_{Iu}$ with respect to CS (*p* = 0.049). Indeed, with ultrasonographic measures, Lee et al. [54] demonstrated that F1 and F2 are strongly correlated to the oral cavity anterior length and the tongue posterior superficial length. Moreover, T0(F0 min) /a/ and T0(F0 max) /a/ show significant statistical differences with respect to HS (*p* ≤ 0.001 and *p* = 0.002, respectively), which could be related to patients’ difficulty in maintaining stable and regular vocal-fold vibration during phonation.

Statistical analysis of FA diagnosed with NS has highlighted differences in F0 mean /a/ and F0 mean /I/ with respect to HS (*p* value = 0.005 and *p* value = 0.028, respectively) and in F0 mean /u/ with respect to CS (*p*-value = 0.006). These alterations might depend on the shorter height and neck with respect to control subjects, a common phenotypical feature for this syndrome. Moreover, jitter /I/ showed a significant difference with respect to CS (*p*-value = 0.018) and SMS (*p*-value = 0.025). However, this consideration must be taken with caution due to the limited size of our database. As shown in Figure 3b, the NS FA vowel triangle is characterized by a small area, but VSA did not show any statistical significance. Nevertheless, formant coordinates have shown significant differences in F2 mean /a/ with respect to HS (*p*-value = 0.001), in F1 mean /u/ with respect to DS (*p*-value = 0.001), and in F2 mean /I/ with respect to SMS (*p*-value = 0.024). Such alterations can be associated with difficulties in lips protrusion and lateralization [37].

Regarding the NS MA group, NNE values were closer to 0, especially for /I/ and /u/ with respect to HS (*p* value < 0.001 and *p* value = 0.001, respectively), which might be associated with the presence of an anterior glottis web [55] or a tendency to incur vocal fold paralysis. However, since this work is mainly focused on acoustical analysis, it was not possible to verify this statement through laryngostroboscopy for these patients. Figure 3c shows vowel-area reduction and centralization. Significant differences in both VSA and FCR were detected with respect to HS (*p* value < 0.001 and *p* value = 0.001, respectively). F2 also showed significant differences: F2 mean /a/ with respect to DS (*p* value < 0.001), F2 mean /I/ with respect to HS (*p*-value = 0.004), and F2 mean /u/ with respect to HS (*p*-value = 0.028). Such alterations might depend on structural properties, such as choanal atresia, supraglottic stenosis, soft palate laxity, and neurologic problems.

In PS SMS subjects, articulation measures and formants showed significant statistical differences for F1 mean /u/ with respect to CS (*p* = 0.034), F2 mean /a/ with respect to HS (*p* = 0.037), F2 mean /I/ with respect to HS (*p* = 0.015) and CS (*p* = 0.001), F ratio${}_{au}$ with respect to HS (*p* = 0.004), and FCR with respect to HS (*p* = 0.001). According to Hidalgo et al. [12], neither F1 nor F2 could discriminate SMS individuals from the control group. This difference could have resulted from the use of different acoustical analysis software tools. First-formant alterations may be linked to velopharyngeal insufficiency, which is an incomplete closure typical of SMS patients that causes a constant airflow leak through nasal cavities, consequently altering resonant frequency along the vocal tract [38].

For the FA group diagnosed with SMS, significant differences were found for F2 mean /a/ with respect to HS (*p*-value = 0.026), F2 mean /I/ with respect to NS (*p*-value = 0.024), and F3 median /I/ with respect to NS (*p*-value = 0.001) and CS (*p*-value = 0.039). Hypotonia and structural lip malformations [11], in addition to frontal lobe calcification and cortical atrophy, could be the reasons for these anomalies.

For the MA SMS group, orofacial dysfunctions worsened by hypotonia, soft-palate clefts, and posterior fossa anomalies might be responsible for articulation disabilities and related to significant differences that were identified for F1 min /a/ and F2 max /u/ with respect to HS (*p* value = 0.050 and *p* value = 0.009, respectively).

The shape and position of the vocalic triangles shown in Figure 3 show that age and gender strongly influence F1 and F2 compared to the reference adult males (solid line with diamond markers); the PS group; and, to a lesser extent, the FA group. Higher formant values are associated with shorter and smaller sizes of the vocal folds and vocal tract. These results underline the importance of conducting acoustical analysis considering age and gender. Moreover, as shown in Figure 3c, a difference also exists between the healthy adult male subjects considered in this study (simple solid line) and the reference adult males (solid line with diamonds), possibly because of our limited sample size.

Table 6 shows an interesting result: HS is always correctly identified in the PS and FA groups. As supported by statistical analysis, the voice quality of normophonic and pathological subjects differs, for allowing an almost complete separation between these two macroclasses.

In particular, the KNN classifier of the PS group achieved the highest mean accuracy of 87%. Such a result was expected due to the larger size of the pediatric subject dataset. The CS class showed a high precision (100%). However, the low recall value (50%), along with its std, suggests that vocal properties might not be specific solely to this syndrome. SMS and NS present a more stable outcome, with especially high specificity scores. However, NS is characterized by a variable recall value (80 ± 42%), which may mean that the NS vocal phenotype is not easy to define.

The SVM model of the FA group performed well on the CS and DS classes. Therefore, these two syndromes seem to present specific voice characteristics, avoiding the other pathologies being classified as NS and CS due to their 100% specificity values. The SMS class showed poor performance, as some NS, CS, and DS observations were classified as SMS, possibly because it was the most numerous class.

Considerations similar to those of the FA group can be applied to the male cohort. High performance characterized CS and DS class recognition as well. It is important to note that the HS were not all correctly identified in the MA group, and some observations were misclassified as DS. The overall accuracy is similar to that of the PS group (84%), but this result must be taken cautiously, as the MA cohort was the smallest in our study. Therefore, in the future, it will be important to understand whether the same performance can be achieved by increasing the sample size and reducing the number of parameters.

These preliminary results are promising in terms of defining a phonatory profile for genetic diseases. However, we remark that this outcome was obtained with a limited dataset, so more voice samples must be collected. Another limitation is the choice to use all available recordings for some of the patients in the case of extremely rare diseases; although precautions were taken, the results might be biased due to the lack of totally independent data. By applying the proposed procedure to a larger dataset, it will be possible to carry out reliable comparisons to validate and possibly find new acoustical features that could reliably describe genetic syndromes. Indeed, with a large amount of data, new models could be developed to determine whether the same differences in the acoustical parameters between syndromes found in this work can be confirmed and whether any improvements in classification results are feasible. For this exploratory analysis, we used acoustical features of the sustained /a/, /I/, and /u/ vowels to obtain results comparable with those reported in previous works. Moreover, these vowels were the most numerous vocal tasks in our small dataset. The small number of subjects analyzed in this first study did not allow for investigation of feature selection or feature engineering techniques to obtain better classifiers. Such methods will be implemented once a more extensive database is available.

## 6. Conclusions

In the present work, acoustical voice analysis of patients affected by genetic syndromes was performed according to a new procedure that can be easily applied in clinical and domestic environments, as it does not require any special equipment. These guidelines allowed us to obtain reliable acoustical parameters and assess voice properties not only by comparing healthy and pathological subjects but also by looking for acoustical differences among the four genetic syndromes considered herein, i.e., Costello, Down, Noonan, and Smith–Magenis syndromes, and with respect to healthy subjects. Acoustical parameters represent an important phenotypical aspect that can be measured non-invasively and, in addition to somatic traits analyzed by dysmorphologists, can help address further medical examinations for diagnosis when genetic screenings are not available or when the syndrome’s genome is still under evaluation. We analyzed these four syndromes considering the results in the literature that demonstrated the presence of neurological and structural problems associated with organs involved in voice production that alter phonation and articulation. The aim of the present paper was to develop an easy, robust, and efficient procedure for analyzing and classifying vocal traits specific to a number of genetic syndromes that can be used to set up and organize a large database. This will help extend existing results, comparing voice production between pathological and healthy subjects to highlight differences and find the best parameters for each syndrome. Through this procedure, we also aim to perform more detailed statistical analyses and implement new artificial intelligence approaches. A larger dataset will allow for further studies to identify which morphological anomalies are linked to altered voice properties and to verify the existence of possible vocal phenotype variability within single syndromes.

## Figures and Tables

**Table 1:** BioVoice and derived acoustical parameters. (${}^{+}$) denotes the parameters used in this study.

Feature	Description
F0 mean (Hz) ${}^{+}$	Mean fundamental frequency
F0 median (Hz) ${}^{+}$	Median fundamental frequency
F0 std (Hz) ${}^{+}$	Standard deviation of the fundamental frequency
F0 min (Hz) ${}^{+}$	Minimum fundamental frequency
T0 (F0 min) (s) ${}^{+}$	Time instant at which the minimum of F0 occurs
F0 max (Hz) ${}^{+}$	Maximum fundamental frequency
T0 (F0 max) (Hz) ${}^{+}$	Time instant at which the maximum of F0 occurs
Jitter (%) ${}^{+}$	Frequency variation of F0
NNE (dB) ${}^{+}$	Normalized noise energy
F1 mean (Hz) ${}^{+}$	Mean value of the first formant
F1 median (Hz) ${}^{+}$	Median value of the first formant
F1 std (Hz) ${}^{+}$	Standard deviation of the first formant
F1 min (Hz) ${}^{+}$	Minimum value of the first formant
F1 max (Hz) ${}^{+}$	Maximum value of the first formant
F2 mean (Hz) ${}^{+}$	Mean value of the second formant
F2 median (Hz) ${}^{+}$	Median value of the second formant
F2 std (Hz) ${}^{+}$	Standard deviation of the second formant
F2 min (Hz) ${}^{+}$	Minimum value of the second formant
F2 max (Hz) ${}^{+}$	Maximum value of the second formant
F3 mean (Hz) ${}^{+}$	Mean value of the third formant
F3 median (Hz) ${}^{+}$	Median value of the third formant
F3 std (Hz) ${}^{+}$	Standard deviation of the third formant
F3 min (Hz) ${}^{+}$	Minimum value of the third formant
F3 max (Hz) ${}^{+}$	Maximum value of the third formant
Signal duration (s)	Total audio file duration
% voiced	Percentage of voiced parts inside the whole signal
Voiced duration (s)	Total duration of voiced parts
Number units	Number of voiced parts
Duration mean (s)	Mean duration of voiced parts
Duration std (s)	Standard deviation of the duration of voiced parts
Duration min (s)	Minimum duration of voiced parts
Duration max (s)	Maximum duration of voiced parts
Number pauses	Total number of pauses in the audio file
Pause duration mean (s)	Mean duration of pauses
Pause duration std (s)	Standard deviation of the duration of pauses
Pause duration min (s)	Minimum duration of pauses
Pause duration max (s)	Maximum duration of pauses
VSA ${}^{+}$	Vowel space area
FCR ${}^{+}$	Formant centralization ratio
F ratio${}_{aI}^{+}$	Formant ratio between F1 of /a/ and F1 of /I/
F ratio${}_{au}^{+}$	Formant ratio between F1 of /a/ and F1 of /u/
F ratio${}_{Iu}^{+}$	Formant ratio between F2 of /I/ and F2 of /u/

**Table 2:** Mean age in years with standard deviation (in parentheses) and number of recordings (in square brackets) in each group.

	PS	FA	MA
CS	9.9 (2.0) [9]	16.4 (4.3) [15]	29.5 (2.1) [6]
DS	7.2 (3.6) [18]	21.2 (11.7) [12]	18.3 (2.2) [9]
NS	10.7 (2.3) [15]	22.4 (7.7) [18]	23.7 (8.4) [18]
SMS	8.0 (2.0) [24]	17.5 (1.3) [15]	16.3 (1.5) [9]
HS	8.9 (3.1) [21]	18.3 (6.8) [9]	21.3 (6.4) [18]

**Table 3:** Statistically significant differences in the acoustical parameters for the PS group. * denotes the difference between one (or more) pathological class and control subjects. ${}^{\#}$ denotes one (or more) difference across genetic syndromes.

Parameter	Kruskal–Wallis H Statistic	*p* Value
F0 std /a/ ${}^{\#}$	11.58	0.021
T0 (F0 min) /a/ *	19.68	<0.001
T0 (F0 max) /a/ *	23.40	<0.001
NNE /a/	11.14	0.025
F1 median /a/ *${}^{\#}$	20.02	<0.001
F1 min /a/ *${}^{\#}$	21.56	<0.001
F1 max /a/ *${}^{\#}$	16.50	0.002
F2 mean /a/ *${}^{\#}$	20.29	<0.001
F2 std /a/ ${}^{\#}$	13.27	0.01
F2 min /a/ *	13.84	0.008
F2 max /a/ *${}^{\#}$	29.77	<0.001
F3 mean /a/ *	10.80	0.029
F3 std /a/ ${}^{\#}$	22.01	<0.001
F3 min /a/ *	10.09	0.039
F3 max /a/ *	15.69	0.003
T0 (F0 min) /I/ *	19.58	<0.001
T0 (F0 max) /I/	10.75	0.03
F2 mean /I/ *${}^{\#}$	20.62	<0.001
F2 max /I/ ${}^{\#}$	17.44	0.002
F1 mean /u/ ${}^{\#}$	10.93	0.027
F1 std /u/ ${}^{\#}$	10.44	0.034
F1 min /u/ ${}^{\#}$	15.70	0.003
F2 std /u/ ${}^{\#}$	14.29	0.006
F2 max /u/ ${}^{\#}$	10.93	0.027
F3 std /u/ ${}^{\#}$	12.80	0.012
F3 max /u/ ${}^{\#}$	10.50	0.033
F ratio${}_{aI}$ *${}^{\#}$	18.14	0.001
F ratio${}_{au}$ *	18.07	0.002
F ratio${}_{Iu}{\;}^{\#}$	11.94	0.018
VSA *${}^{\#}$	17.53	0.002
FCR *${}^{\#}$	26.98	<0.001

**Table 4:** Statistically significant differences in the acoustical parameters for the FA group. * denotes the difference between one (or more) pathological class and control subjects. ${}^{\#}$ denotes one (or more) difference across genetic syndromes.

Parameter	Kruskal–Wallis H Statistic	*p* Value
F0 mean /a/ *${}^{\#}$	18.70	<0.001
F0 min /a/ ${}^{\#}$	14.76	0.005
F0 max /a/ *${}^{\#}$	17.37	0.002
NNE /a/	11.50	0.022
F1 mean /a/	14.07	0.007
F1 std /a/ *${}^{\#}$	18.53	<0.001
F1 min /a/ *${}^{\#}$	18.14	0.001
F2 mean /a/ *	19.01	<0.001
F2 std /a/ *${}^{\#}$	16.00	0.003
F2 min /a/	10.20	0.04
F2 max /a/ *${}^{\#}$	24.78	<0.001
F0 mean /I/ *${}^{\#}$	18.70	<0.001
F0 std /I/ ${}^{\#}$	11.07	0.026
F0 min /I/ *${}^{\#}$	13.05	0.011
F0 max /I/ *${}^{\#}$	19.55	<0.001
Jitter /I/ ${}^{\#}$	21.09	<0.001
NNE /I/ ${}^{\#}$	10.41	0.034
F1 std /I/ ${}^{\#}$	15.94	0.003
F1 min /I/ ${}^{\#}$	13.07	0.011
F2 mean /I/ ${}^{\#}$	14.13	0.007
F2 std /I/ ${}^{\#}$	12.57	0.014
F2 min /I/ ${}^{\#}$	15.65	0.004
F3 mean /I/ ${}^{\#}$	17.60	0.001
F3 min /I/ ${}^{\#}$	14.07	0.007
F3 max /I/ ${}^{\#}$	15.14	0.004
F0 mean /u/ ${}^{\#}$	17.24	0.002
F0 std /u/ ${}^{\#}$	12.73	0.013
F0 min /u/ *${}^{\#}$	19.72	<0.001
F0 max /u/ ${}^{\#}$	11.87	0.018
Jitter /u/ *${}^{\#}$	11.77	0.019
F1 mean /u/ ${}^{\#}$	17.38	0.002
F1 min /u/ ${}^{\#}$	17.77	0.001
F2 mean /u/ *	13.89	0.008
F2 std /u/ ${}^{\#}$	14.65	0.005
F2 min /u/ *${}^{\#}$	13.38	0.01
F2 max /u/ *${}^{\#}$	17.54	0.002
F3 std /u/	10.50	0.033

**Table 5:** Statistically significant difference in the acoustical parameters for the MA group. * denotes the difference between one (or more) pathological class and control subjects. ${}^{\#}$ denotes one (or more) difference across genetic syndromes.

Parameter	Kruskal–Wallis H Statistic	*p* Value
F0 mean /a/ *	22.61	<0.001
F0 min /a/ *	21.28	<0.001
F0 max /a/ *	22.29	<0.001
F1 std /a/ *${}^{\#}$	23.17	<0.001
F1 min /a/ *	14.70	0.005
F2 mean /a/ *${}^{\#}$	20.67	<0.001
F2 min /a/ *${}^{\#}$	29.86	<0.001
F2 max /a/ *${}^{\#}$	15.38	0.004
F3 mean /a/ *${}^{\#}$	19.49	<0.001
F3 min /a/ *${}^{\#}$	18.36	0.001
F3 max /a/ *${}^{\#}$	18.19	0.001
F0 mean /I/ *	18.31	0.001
F0 max /I/ *	21.74	<0.001
NNE /I/ *	24.75	<0.001
F2 mean /I/ *	15.58	0.004
F2 std /I/ *	13.60	0.009
F2 min /I/ *	16.94	0.002
F2 max /I/ *	11.81	0.019
F0 mean /u/ *${}^{\#}$	25.06	<0.001
T0(F0 min) /u/ *${}^{\#}$	15.99	0.003
F0 max /u/ *${}^{\#}$	24.86	<0.001
NNE /u/ *	16.51	0.002
F1 mean /u/ *	11.67	0.02
F1 std /u/ *	17.51	0.002
F1 min /u/ ${}^{\#}$	14.64	0.006
F1 max /u/ *	12.66	0.013
F2 mean /u/ *	16.32	0.003
F2 min /u/	10.08	0.039
F2 max /u/ *	27.40	<0.001
F3 mean /u/	11.58	0.021
F3 std /u/ *	12.71	0.013
F3 min /u/ *	18.99	<0.001
F ratio${}_{au}$ *	10.27	0.036
F ratio${}_{Iu}$ *	23.07	<0.001
VSA *	22.82	<0.001
FCR *	19.33	<0.001

**Table 6:** Performance of the best classifiers for the PS, FA, and MA groups. Mean value ± std.

PS					
**Parameter**	**SMS**	**NS**	**CS**	**DS**	**HS**
Precision	88 ± 19%	88 ± 23%	100 ± 0%	93 ± 14%	100 ± 0%
Recall	100 ± 0%	80 ± 42%	50 ± 53%	100 ± 0%	100 ± 0%
Specificity	92 ± 14%	96 ± 10%	100 ± 0%	95 ± 11%	100 ± 0%
F1 Score	93 ± 12%	92 ± 15%	100 ± 24%	96 ± 8%	100 ± 0%
AUC	99 ± 1%	97 ± 3%	85 ± 4%	81 ± 10%	99 ± 0%
Validation Accuracy	87 ± 9%				
**FA**					
**Parameter**	**SMS**	**NS**	**CS**	**DS**	**HS**
Precision	60 ± 33%	88 ± 34%	100 ± 0%	100 ± 0%	100 ± 0%
Recall	90 ± 32%	70 ± 48%	60 ± 52%	90 ± 32%	100 ± 0%
Specificity	70 ± 23%	100 ± 11%	100 ± 0%	100 ± 0%	100 ± 0%
F1 Score	77 ± 19%	100 ± 0%	100 ± 0%	100 ± 0%	100 ± 0%
AUC	95 ± 5%	96 ± 2%	79 ± 9%	93 ± 8%	100 ± 0%
Validation Accuracy	77 ± 19%				
**MA**					
**Parameter**	**SMS**	**NS**	**CS**	**DS**	**HS**
Precision	78 ± 36%	89 ± 33%	94 ± 17%	100 ± 0%	100 ± 0%
Recall	89 ± 33%	89 ± 33%	90 ± 32%	80 ± 42%	80 ± 30%
Specificity	88 ± 19%	97 ± 11%	95 ± 16%	100 ± 0%	100 ± 0%
F1 Score	92 ± 15%	100 ± 0%	96 ± 11%	100 ± 0%	87 ± 17%
AUC	93 ± 5%	97 ± 2%	100 ± 0%	100 ± 0%	94 ± 5%
Validation Accuracy	84 ± 17%				

**Figure A1:**
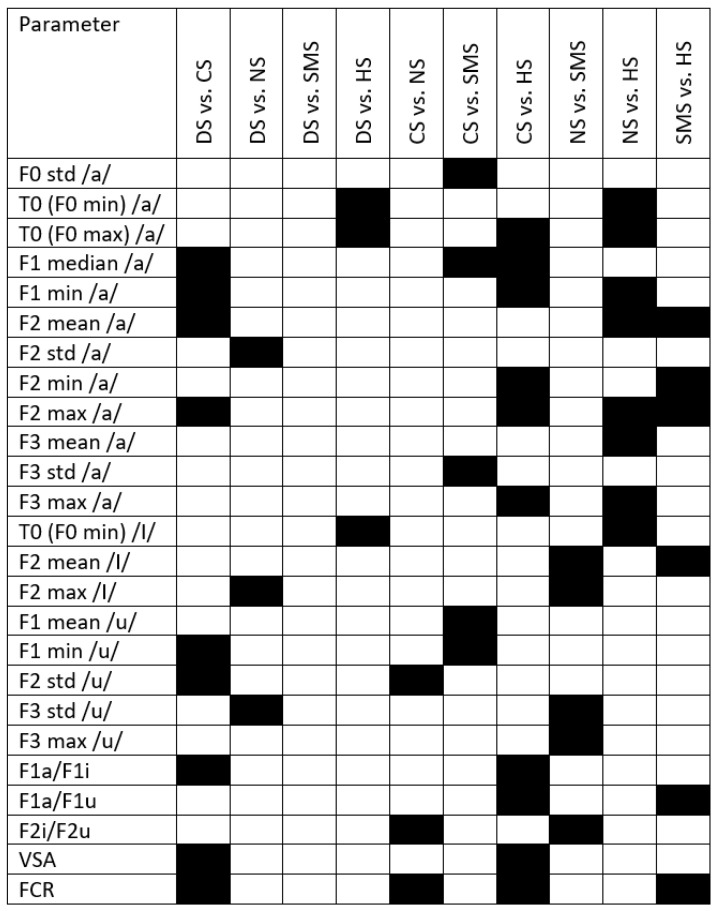
Black boxes refer to significant differences between syndromes and with respect to healthy subjects. DS = Down syndrome; CS = Costello syndrome; NS: Noonan syndrome; SMS = Smith–Magenis syndrome; HS = healthy subjects; VSA = vowel space area; FCR = formant centralization ratio.

**Figure A2:**
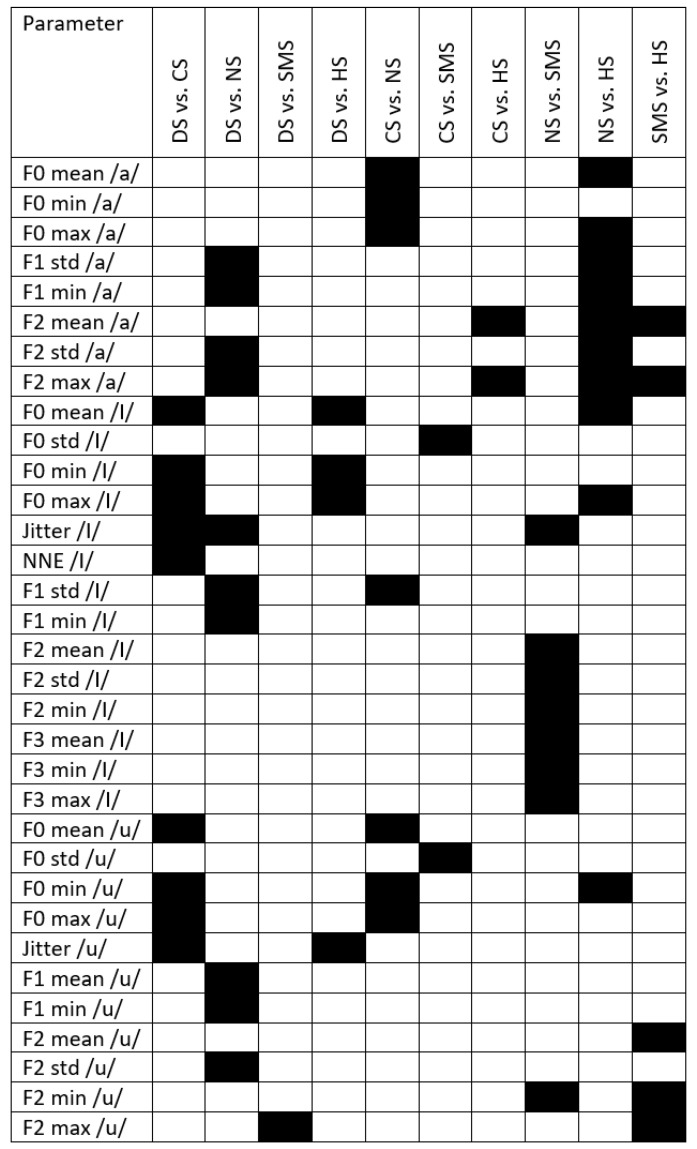
Black boxes refer to significant differences between syndromes and with respect to healthy subjects. DS = Down syndrome; CS = Costello syndrome; NS: Noonan syndrome; SMS = Smith–Magenis syndrome; HS = healthy subjects; VSA = Vowel space area; FCR = formant centralization ratio.

**Figure A3:**
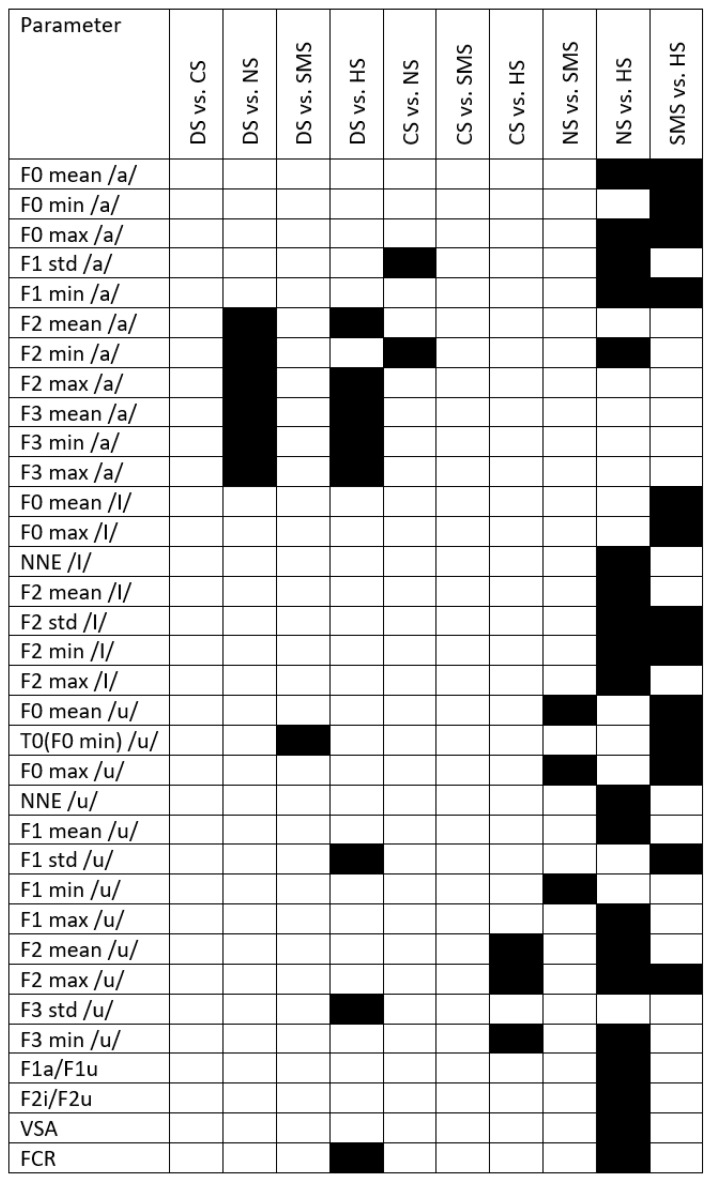
Black boxes refer to significant differences between syndromes and with respect to healthy subjects. DS = Down syndrome; CS = Costello syndrome; NS: Noonan syndrome; SMS = Smith–Magenis syndrome; HS = healthy subjects; VSA = vowel space area; FCR = formant centralization ratio.

## Data Availability

Data are available from the corresponding author upon request.

## Appendix A

Figure A1 displays the results of post hoc analysis for the PS group. Black boxes highlight significant differences between groups.

## Appendix B

Figure A2 displays the results of post hoc analysis for the FA group. Black boxes highlight significant differences between groups.

## Appendix C

Figure A3 displays the results of post hoc analysis for the MA group. Black boxes highlight significant differences between groups.
